# Moisture content online detection system based on multi-sensor fusion and convolutional neural network

**DOI:** 10.3389/fpls.2024.1289783

**Published:** 2024-03-04

**Authors:** Taoqing Yang, Xia Zheng, Hongwei Xiao, Chunhui Shan, Jikai Zhang

**Affiliations:** ^1^ College of Mechanical and Electrical Engineering, Shihezi University, Shihezi, China; ^2^ Key Laboratory of Northwest Agricultural Equipment, Ministry of Agriculture and Rural Affairs, Shihezi, China; ^3^ Key Laboratory of Modern Agricultural Machinery Corps, Shihezi, China; ^4^ College of Engineering, China Agricultural University, Beijing, China; ^5^ College of Food, Shihezi University, Shihezi, China

**Keywords:** convolutional neural network, prediction model, multi-sensor fusion, moisture content, online detection

## Abstract

To monitor the moisture content of agricultural products in the drying process in real time, this study applied a model combining multi-sensor fusion and convolutional neural network (CNN) to moisture content online detection. This study built a multi-sensor data acquisition platform and established a CNN prediction model with the raw monitoring data of load sensor, air velocity sensor, temperature sensor, and the tray position as input and the weight of the material as output. The model’s predictive performance was compared with that of the linear partial least squares regression (PLSR) and nonlinear support vector machine (SVM) models. A moisture content online detection system was established based on this model. Results of the model performance comparison showed that the CNN prediction model had the optimal prediction effect, with the determination coefficient (*R^2^*) and root mean square error (RMSE) of 0.9989 and 6.9, respectively, which were significantly better than those of the other two models. Results of validation experiments showed that the detection system met the requirements of moisture content online detection in the drying process of agricultural products. The *R^2^* and RMSE were 0.9901 and 1.47, respectively, indicating the good performance of the model combining multi-sensor fusion and CNN in moisture content online detection for agricultural products in the drying process. The moisture content online detection system established in this study is of great significance for researching new drying processes and realizing the intelligent development of drying equipment. It also provides a reference for online detection of other indexes in the drying process of agricultural products.

## 1 Introduction

As an essential parameter in the drying processing of agricultural products, moisture content characterizes the drying rate and signals the end of drying (Yu et al., 2023). Achieving online detection of moisture content in the drying process is essential to optimize the drying process and realize the automation of drying. At present, material moisture content online detection methods include the dielectric properties method (Celik et al., 2022), model prediction method (Dalvi-Isfahan, 2020), spectral imaging method (Cho et al., 2020), and weighing method (Pongsuttiyakorn et al., 2019). The dielectric property method is a moisture content detection method based on the correlation between the dielectric properties of the material and the moisture content. The dielectric properties of the material are greatly affected by temperature, and performing accurate moisture content detection when the material is dried at different temperatures is not easy. The model prediction method is suitable for moisture content detection of specific materials under a specific drying environment. When the material or drying environment changes, the model needs to be re-established to detect moisture content. The spectral imaging method is expensive and requires computer vision technology, which is complicated to operate, not applicable to the agricultural product drying industry with low added value. The weighing method can detect the moisture content of different materials with high versatility, low cost, and simple operation, and is an essential method of moisture content online detection.

The weighing method is a method for real-time acquisition of the weight of the material during the drying process, according to the principle of constant dry matter, combined with the initial moisture content of the material to achieve moisture content online detection. The key to the weighing method is accurately acquiring the material weight by using the load sensor. The complex drying environment, the vibration of equipment, the impact and disturbance of airflow, and the variation of drying temperature will bring severe errors to the detection of the load sensor, which will affect the accuracy of the moisture content detection. Ju et al. (2023) stopped the blower to avoid airflow’s influence on the load sensor’s detection during moisture content detection but ignored the error caused by temperature variation. Yang et al. (2023a) similarly achieved moisture content detection by using the stop-air detection strategy and corrected the detection error caused by temperature change, improving moisture content detection accuracy. However, in different drying programs, the temperature variation range is far beyond the linear calibration interval of the load sensor, and achieving accurate measurement by simply compensating the error due to temperature change is difficult. Wang et al. (2014) while using a stop-air detection strategy at the same time, carried out linearization calibration of the detection results of the load sensor at different temperature sections and load ranges. The scheme effectively avoids the influence of temperature on the detection of the load sensor. Reyer et al. (2022) directly installed the load sensor in the drying chamber outside, more effectively eliminating the measurement error caused by temperature. However, this scheme destroyed the sealing of the drying chamber, which increased the difficulty of controlling the temperature and humidity in the drying chamber. The above moisture content online detection scheme was implemented under the stop-air detection strategy.

With the development of automation and intelligence in the drying industry, drying equipment needs to make real-time adjustments to the temperature and humidity in the drying chamber according to the drying rate, and it needs to detect the moisture content more frequently. In this context, stopping the blower to detect moisture content will undoubtedly break the continuity of drying and further increase energy consumption and drying time. Therefore, the existing moisture content online detection technology cannot meet the needs of the current drying process.

Multi-sensor fusion technology is an information processing method that uses computer technology to automatically analyze and synthesize information and data from multiple sensors or sources under specific guidelines to obtain the required decisions and estimates (Xie et al., 2022). Factors affecting load sensor detection, such as vibration of equipment, impact and disturbance of airflow, and temperature variation, can be detected by the sensors. Multi-sensor fusion technology can fuse the load sensor signal with other sensor signals, make regression prediction of the real weight of the material in the drying process, and further detect the moisture content of the material. Regression prediction based on multi-sensor fusion technology has been widely used in other industries, such as the remaining life prediction of aviation engines (Li et al., 2022b), tool wear prediction (Meng et al., 2021), air pollution level prediction (Ari and Alagoz, 2022), and wheel odometry prediction (Zhu et al., 2021). Kirsanov et al. (2021) used PLSR to relate the sensor signals to the values of different water quality parameters, which enabled the accurate detection of various water quality parameters. Li et al. (2019) has applied SVM in multi-sensor fusion to assess green tea quality accurately.

The complex dry environment causes all kinds of sensor signals to fluctuate and behave randomly. Raw sensor signals are difficult to transform into a stable output value after filtering. At the same time, the filtering process removes essential information hidden in the raw signals that are correlated with the output. Deep learning has been introduced into multi-sensor fusion prediction to obtain the correlation and causality hidden in raw monitoring data (Xu et al., 2020). Deep learning is a specific machine learning type consisting of a stack of multilayer nonlinear processing units (Samaras et al., 2019). Deep learning techniques have more powerful representational learning capabilities than traditional machine learning techniques. They can learn complex functions that map inputs to outputs directly from raw data (Wang et al., 2021). Convolutional neural network (CNN), a class of feed-forward neural networks that include convolutional computation and have a deep structure, are one of the representative algorithms for deep learning (Tong et al., 2023). CNN have also been widely used in solving regression prediction problems with multi-sensor fusion and have contributed to many tasks with state-of-the-art accuracy (Arvidsson et al., 2021; Zeng et al., 2021; Wan et al., 2022; Li et al., 2022a; Gao et al., 2023).

Given the air-impingement dryer’s fast drying speed and high heat transfer coefficient, this study built a moisture content online detection system in the air-impingement dryer (Yang et al., 2023c). The tray position needs to be added to the prediction model as an input variable because of the particularity of the structure of the air-impingement dryer. Overall, this study applied multi-sensor fusion technology to the moisture content online detection process and used the CNN prediction model to fuse the raw signals from the weight sensor, air velocity sensor, temperature sensor, and the tray position to accurately obtain the real weight of the material in the drying process. According to the initial moisture content of the material, the current moisture content was obtained, and finally, the regression prediction model of moisture content was established. A moisture content online detection system was built based on this model.

**Figure 1:**
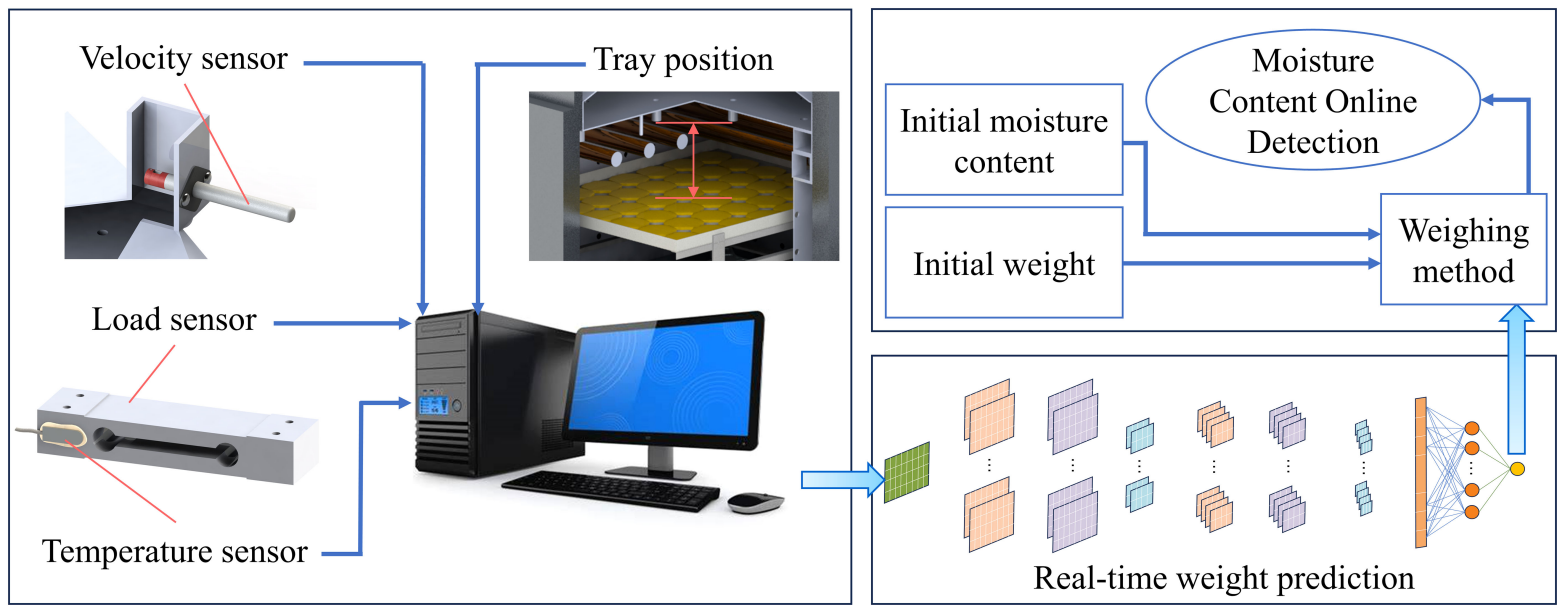
Technology Roadmap.

In summary, this study (1) completed the construction of a multi-sensor data acquisition platform; (2) carried out cantaloupe slice drying experiments to obtain the raw monitoring signals of multi-sensors used for CNN training; (3) established a material weight prediction model based on CNN and compared it with the traditional prediction model; and (4) established a moisture content online detection system based on the CNN prediction model. The technology roadmap is shown in **Figure 1**. This study built a moisture content online detection system and will provide new technical support for drying process optimization and promote the intelligent development of drying equipment.

## 2 Principles and methods

### 2.1 Principles

In this study, the online detection system was built in the air-impingement dryer and realized the online detection of the material moisture content based on the weighing method. The following sections show the operating principle of air-impingement dryer and the principle of moisture content detection based on the weighing method.

#### 2.1.1 Operation principle of air-impingement dryer

**Figure 2:**
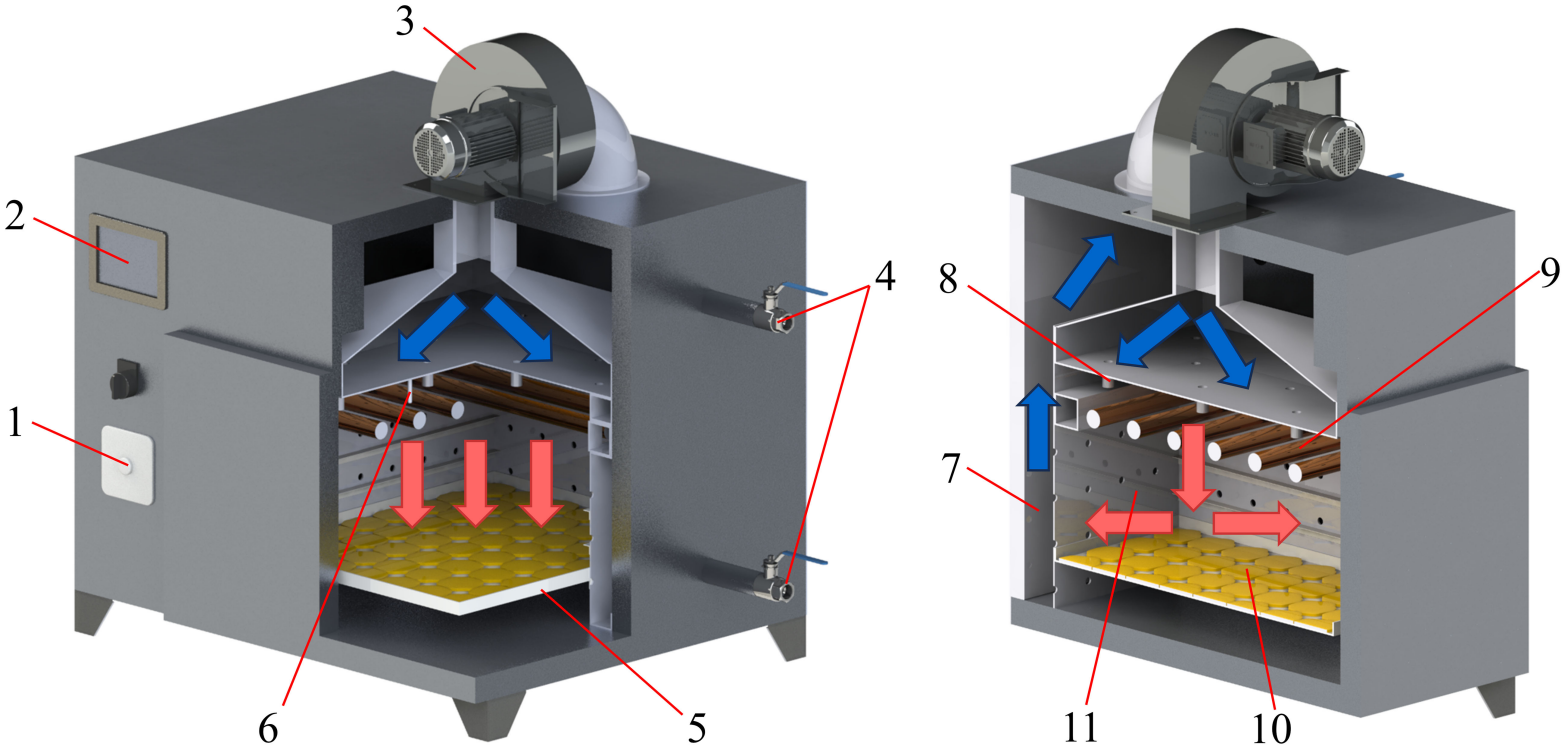
Operation principle diagram of air-impingement dryer. (1) Air velocity adjustment knob; (2) temperature control touch panel; (3) fan; (4) wet discharge valve; (5) tray; (6) temperature sensor; (7) outer chamber; (8) air nozzle; (9) infrared heating tube; (10) material; (11) inner chamber.

The air-impingement dryer is a technology that realizes drying by impinging and heating the material with pressurized hot air (Zheng et al., 2023). **Figure 2** shows the operation principle diagram of the air-impingement dryer. The air-impingement dryer is divided into the inner chamber and the outer chamber. Six infrared heating tubes are evenly installed on the top of the inner chamber, with a total power of 0–2 KW. The infrared heating tubes heat the materials placed on the tray with infrared radiation. The fan draws air from the inner chamber into the outer chamber. The air is cooled in the outer chamber, and the wet air is discharged from the outer chamber through a wet discharge valve. The fan blows the air into the inner chamber through the nozzle to realize internal circulation of the air in the equipment. When the air through the nozzle is squeezed, it forms a high-pressure airflow and impacts the material, removing the moisture on its surface. The material is dried under the double effect of infrared radiation heating and airflow impact.

The dryer regulates the air velocity of the fan through a frequency converter. The dryer is not equipped with an air velocity sensor, which cannot achieve closed-loop regulation of the air velocity, so there are large fluctuations in the airflow in the inner chamber. A temperature sensor is installed at the nozzle, which is used to detect the temperature of the air in the inner chamber. The equipment achieves closed-loop control of the air temperature in the inner chamber by adjusting the power of the infrared heating tube. The temperature of the outer chamber is significantly lower than that of the inner chamber due to the lack of heating by the infrared heater. The internal circulation of air increases the difficulty of temperature control in the inner chamber.

#### 2.1.2 Principle of moisture content detection based on the weighing method

Moisture content detection based on the weighing method is a method to calculate the moisture content based on the initial weight and the real-time weight during the drying process under the default condition that the initial moisture content of the same batch of material is the same. The formula for calculating the moisture content based on the weighing method (wet basis) is shown in Equation 1 (Liu et al., 2021):


(1) $$w_{t}=\frac{m_{t}-m\left(1-w_{i}\right)}{m_{t}}\times 100\%$$

where *w_t_* is the moisture content (wet base) of the material at time *t*, %; *m_t_* is the weight of the material at time *t*, g; *m* is the initial weight of the material, g; and *w_i_* is the initial moisture content (wet basis) of the material, %.

A load sensor usually needs to be installed at the bottom of the rack to obtain the weight of the material at time *t*. During the actual drying process, the load sensor has difficulty outputting a stable weight signal due to airflow disturbances and equipment vibration. The impact of airflow and temperature variation also causes measurement errors in the load sensor. During the air-impingement drying process, people often change the tray position on the rack to obtain different drying quality and drying rates of the material (Chang et al., 2022). Preliminary experiments found that the tray position also significantly affects the load sensor’s measurement results. The tray position here indicates the distance between the tray and the nozzle.

The drying temperature, air velocity, tray position, and material weight set by the drying process of different materials vary greatly. Therefore, the error caused by the complex dry environment to the detection value of the load sensor needs to be eliminated. In addition to the tray position, other influencing factors can be detected by the sensor. The air velocity sensor can detect the airflow speed, and its raw signal can also reflect the airflow fluctuation. The temperature sensor can detect the temperature value that affects the measurement value of the load sensor. The device’s vibration will also be reflected in the raw signal of the load sensor.

### 2.2 Multi-sensor data acquisition

The monitoring data from the three sensors during the drying process need to be collected for model training to establish a moisture content online detection model with the raw signals from load sensor, air velocity sensor, temperature sensor, and the tray position as inputs and the real weight of the material as outputs.

#### 2.2.1 Multi-sensor data acquisition platform construction

**Figure 3:**
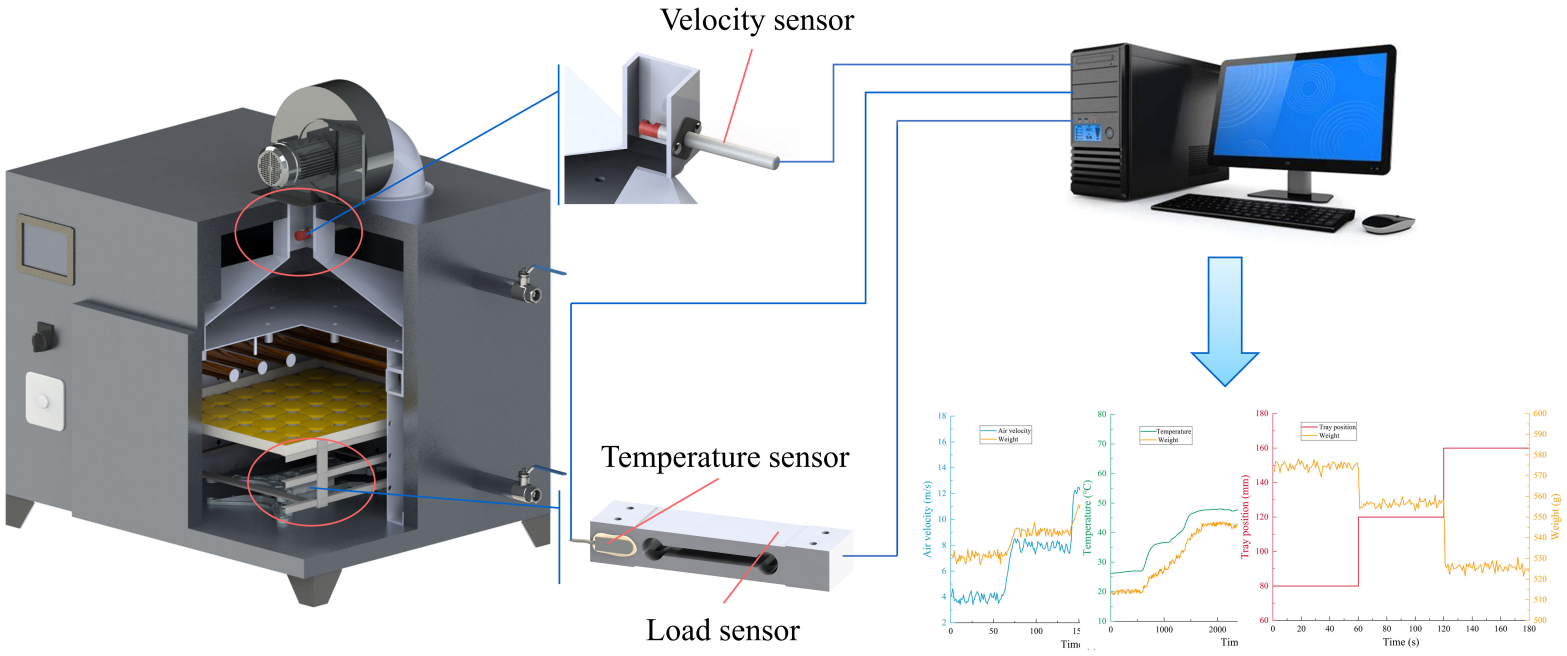
Multi-sensor data acquisition system.

The data acquisition system consists of an upper computer, a weight acquisition module, an air velocity acquisition module, a temperature acquisition module, and a 485 communication module as shown in **Figure 3**. The upper computer adopted the Legion Y7000P computer from Lenovo, which was responsible for human–computer interaction and data storage. The upper computer adopted the MODBUS communication protocol and connected with each slave unit via three RS485 buses to form a data acquisition network. In the weight acquisition module, the cantilever beam pressure sensor (HYPX017, Hengyuan Sensor Technology Co., Ltd., Bengbu, China) with a range of 3 kg was selected to collect the weight signal of the material in the drying process. In the air velocity acquisition module, a thermal air velocity sensor (WM4200, Chaozhi Reed Technology Co., Ltd., Changchun, China) with a range of 20 m/s was used to acquire the air velocity. The air velocity sensor was installed in the air duct of the outer chamber with a lower temperature to increase the service life of the air velocity sensor and to reduce the influence of temperature on the measurement results of the air velocity sensor. The dimensions of the air duct were 60mm × 50mm. The dimensions of the tray were 400mm × 350mm. Temperature variations in the elastic substrate of the load sensor are the leading cause of measurement errors. In the temperature acquisition module, a temperature sensor (PT100, Songdao Heating Sensor Co., Ltd., Shanghai, China) with a range of −45°C to 125°C was selected to collect the temperature signal of the load sensor elastic substrate. The temperature sensor was fixed to the elastic substrate by using thermally conductive silicone. A 485 communication module was used to communicate between the three sensors and the upper computer. The signals of each sensor were not filtered to obtain the correlation hidden in the raw monitoring data of the sensors.

#### 2.2.2 Design of single-factor experiment

A single-factor experiment was carried out to investigate the effect of air velocity, temperature, and tray position on the measured data from the load sensor. First, air velocity was used as a single-factor variable for the experimental design. The load sensor measurement data were obtained continuously under a constant load of 500 g with a sampling interval of 1 s and duration of 300 s, load sensor substrate temperature of 25°C, tray position of 80 mm, and air velocity varying in the range of 4–16 m/s. The load sensor substrate temperature was set to a zero point temperature of 25°C. The zero point temperature of the load sensor refers to the temperature at which the output voltage of the load sensor is zero at no load, and the weighing value at this temperature is the standard value. In the experiment with load sensor substrate temperature as the single factor variable, the fan stopped running, the constant load was 500 g, the tray position was 80 mm, the temperature varied in the range of 25°C–70°C, the sampling interval was 10 s, and the sampling duration was 80 min. In the experiment with tray position as the single factor variable, the constant load was 500 g, the load sensor substrate temperature was 25°C, and the air velocity was set at 16 m/s. The load sensor data were collected at 80, 120, and 160 mm tray positions with a sampling interval of 1 s and a sampling duration of 180 s.

#### 2.2.3 Experimental design for multi-sensor data acquisition

The data acquisition experiments were carried out under different drying environments to thoroughly investigate the correlation between the input variables and the weight of the material and improve the prediction model’s accuracy. The temperature setting range in the drying of agricultural products is usually 40°C–70°C. The maximum air velocity of the outer air duct in the air-impingement dryer is 16 m/s. The tray position is determined by the structure of the rack, which has three layers in total, and the distances between the tray and the nozzle are 80, 120, and 160 mm, respectively. In summary, the data acquisition experiments were carried out at different temperatures (40°C, 50°C, 60°C, and 70°C), different air velocities (4, 8, 12, and 16 m/s), and different tray positions (80, 120, and 160 mm). Each group drying experiment randomly obtained 10 groups of data, and each group of data sampling interval was greater than 5 minutes, thus obtaining a total of 480 groups of data (4 × 4 × 3 × 10). Each sampling time lasted 8 s, and the sampling frequency was 8 Hz.

Data acquisition experiments were conducted during the cantaloupe slice drying experiment. Fresh, undamaged cantaloupe was peeled, deseeded, and sliced into 30 × 50 × 7 mm slices. For each set of experiments, 1000 g of cantaloupe slices were weighed and placed on the tray. The cantaloupe slices were removed from the tray, weighed immediately after each data acquisition, and quickly returned to the tray. The weight of the cantaloupe slices was the output value of this dataset.

### 2.3 Prediction model of moisture content online detection

#### 2.3.1 Convolutional neural network

CNN is a deep learning model or a multilayer perceptron similar to artificial neural network. In this study, the CNN was used for regression analysis to mine potential information in the raw monitoring data of load sensor, air velocity sensor, temperature sensor, and tray position to achieve weight prediction and complete the study of moisture content online detection.

**Figure 4:**
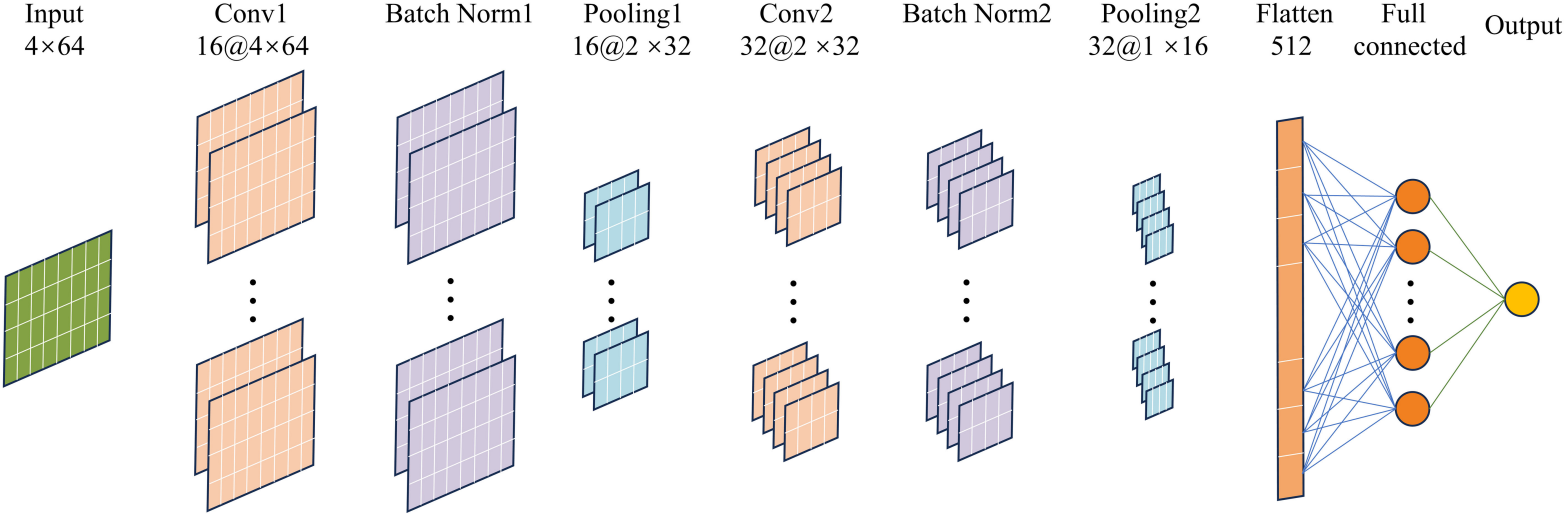
Structure of CNN.

The CNN used in this study consisted of the input layer, convolutional layer, batch normalization layer, average pooling layer, fully connected layer, and output layer, and its structure is shown in **Figure 4**. The function of the input layer was mainly to normalize the input data, which can improve the model’s generalization ability and increase the training speed (Hu et al., 2023). The convolutional layer uses convolutional operations to filter out redundant information in the original data, enhance the information related to the output, and achieve automatic feature extraction (Wang et al., 2022). The convolution kernel size was set to 3 × 3, the convolution mode was set to “same,” and the step size was set to 1. The number of convolution kernels needed to be adapted to the structure of the training data, which was determined by a trial-and-error method based on the performance evaluation index of the model (Ma et al., 2023). The activation function in the neural network structure can make a nonlinear mapping of the output, which is particularly important for the accuracy of the prediction model (Guan et al., 2022).

The CNN model was trained using the rectified linear unit (ReLU) function, the hyperbolic tangent (tanh) function, and the sigmoid function to select the best activation function. The most appropriate activation function was selected based on the model performance evaluation index. The average pooling layer was located after the convolutional layer, and its function was to accomplish the parameter degradation and maintain translation invariant properties, which can be achieved to reduce the feature map while preserving the critical features in the input to some extent (Zhong et al., 2023). The size of the average pooling matrix was set to 2, and the step size was set to 2. Adding a batch normalization layer between the convolutional layer and the average pooling layer allowed the inputs of each neural network layer to maintain the same distribution during neural network training, thus reducing the internal covariate shift, improving the gradient mobility, and achieving the regularization effect (Tan et al., 2021). A dropout layer was set before the fully connected layer. In the dropout layer, some input elements were randomly changed to zero with a probability set to 0.05. The dropout layer randomly rendered 5% of the elements non-functional, thus avoiding overfitting (Zhao et al., 2023). The fully connected layer flattened the feature map into a one-dimensional vector for final feature integration and output prediction. The role of the output layer was to output the predicted result, which in this study was the real weight of the material.

A total of 480 sets of data were randomly sorted, and 70% of the data (336 sets) were used as the training set, 15% of the data (72 sets) were used as the validation set, and 15% of the data (72 sets) were used as the test set. During the training of the CNN, the network parameters were updated according to the loss function for each training batch, and the batch size was set to 16. The training set had 336 sets of data, and one iteration was completed for every 21 updates of the network parameters. The maximum number of iterations was set to 50. The root mean square error (RMSE) between the real values and the predicted values of the validation set was used as the loss function, which was calculated by Equation 2. Model training was performed in a Legion Y7000P computer from Lenovo with MATLAB R2021a software.


(2) $$\text{Loss}=\text{RMSE}=\sqrt{\frac{\sum_{i=1}^{n}{\left(\hat{y_{i}}-y_{i}\right)}^{2}}{n}}\;$$

Where *n* is the number of samples in the validation set, $\hat{y_{i}}$ and *y_i_* are the predicted value and real value of the *i^th^* sample.

#### 2.3.2 Evaluation of model performance

The performance of the prediction model was evaluated in terms of the RMSE of the training set (RMSE_Tr_), validation set (RMSE_Ve_), and test set (RMSE_Te_), and the coefficients of determination (*R^2^*) of the training set (*R^2^_Tr_*), validation set (*R^2^_Ve_*), and test set (*R^2^_Te_*). RMSE and *R^2^* represent the deviation and degree of fitting between the real and predicted values, respectively. RMSE focuses on the magnitude of the error, with smaller values indicating greater accuracy of the model. *R^2^* focuses on the ability of the model to explain the variation in the data, with values closer to 1 indicating a better fit of the model. These evaluation parameters were calculated by Equation 3 and Equation 4 (Wang et al., 2023):


(3) $$R_{Tr}^{2},R_{Ve}^{2},R_{Te}^{2}=1-\frac{\sum_{i=1}^{n}{\left(\hat{y_{i}-}y_{i}\right)}^{2}}{\sum_{i=1}^{n}{\left(y_{i}-y_{m}\right)}^{2}}$$

(4) $${\text{RMSE}}_{\text{Tr}},{\text{RMSE}}_{\text{Ve}},{\text{RMSE}}_{\text{Te}}=\sqrt{\frac{\sum_{i=1}^{n}{\left(\hat{y_{i}}-y_{i}\right)}^{2}}{n}}\;$$

Where *n* is the number of samples in the corresponding set (training set, validation set, and test set), $\hat{y_{i}}$ and *y_i_* are the predicted value and real value of the *i^th^* sample, and *y_m_* is the mean value of all the samples.

### 2.4 System validation experiments

MATLAB software was used for data processing, model prediction, and real-time display of moisture content in the moisture content online detection system. First, the initial weight and the initial moisture content of the material were set, and the initial moisture content was measured by the oven method (Yang et al., 2023b). The sensor cannot detect the tray position and is a fixed value. Thus, this value also needs to be input into the software. MATLAB processed the data obtained from the sensors to meet the format requirements of the predictive model inputs. The processed data were then fed into a trained prediction model, which outputted the real weight of the material. The moisture content of the material was calculated according to the initial weight and the initial moisture content and displayed in real time.

The cantaloupe slice drying experiment in Section 2.2.3 was repeated. The initial weight of cantaloupe slices was 1000 g, and the initial moisture content was 90.19% (wet basis). The set values of air velocity, temperature and tray position were randomized into five experimental groups. The experimental design is shown in **Table 1**. Where temperature refers to the air temperature in the inner chamber, measured by the temperature sensor in **Figure 2**. Five sets of experiments were conducted sequentially under the same set of material conditions, with each set lasting 30 minutes. Three sensors, including a load sensor, an air velocity sensor, and a temperature sensor acquired data once at a random time during each set of test cycles.

**Table 1:** The design of system validation experiments.

Factor	Group				
	1	2	3	4	5
Air velocity (m/s)	12.6	6.3	8.4	14.1	9.7
Tray position (mm)	140	100	140	100	60
Temperature (°C)	68	43	50	56	63

## 3 Results and discussion

### 3.1 Results and analyses of single-factor experiment

**Figure 5:**
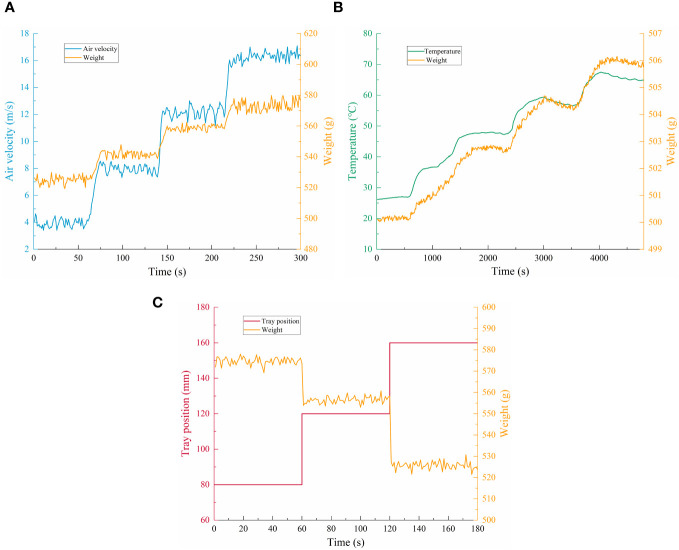
The results of single-factor experiment. **(A–C)** are the experimental results with air velocity, temperature and tray position as single factors, respectively.

**Figure 5A** shows the experimental results with air velocity as a single factor variable. The monitoring signal of the load sensor fluctuated greatly with more noise, which was due to the unstable impact force of the airflow on the tray caused by the inhomogeneity of the airflow. The vibration generated by the equipment operation made the load sensor unable to acquire the data in a stable state. The measured values of the load sensor in different air velocities had a significant difference, and the variation range of the measured values was from 519.34 g to 579.78 g, with a variation of 60.44 g. The wind direction was perpendicular to the tray’s upper surface; thus, the load sensor’s measured values showed a positive relationship with the air velocity and the relationship had a strong transient nature.

**Figure 5B** shows the experimental results with temperature as a single factor variable. The fluctuation range of the load sensor measurement value was 500.03–506.15 g with a fluctuation amplitude of 6.12 g in the temperature variation range of 26.26°C–65.76°C. The monitoring value of the load sensor and the temperature of the load sensor elastic substrate at a fixed load showed a positive relationship. The load sensor used for moisture content detection was a resistance strain gauge pressure sensor. Temperature can cause errors and noise in the measured values of the load sensor by affecting the resistance strain gauges’ resistance value and the elastic substrate’s elastic modulus (Burnos and Rys, 2017). The effect of temperature on the measured value of weight sensors also had significant relationships with the load. Wang et al. (2014) calibrated the measured values of weight sensors at different temperature sections and load ranges, effectively avoiding the influence of temperature on the detection of the load sensor.

**Figure 5C** shows the experiment results with the tray position as a single factor variable. Under a constant load, the tray position significantly affected the load sensor’s measurement value. The fluctuation range of the load sensor measurements at the three tray positions was 521.44–578.01 g, with a fluctuation range of 56.57 g. The smaller the distance between the tray and the nozzles, the more concentrated the airflow from the nozzles, and the greater the force exerted on the tray, which in turn increased the load sensor measurements. The three layers of the tray were arranged vertically so that the tray position did not affect the measured value of the load sensor when the fan was stopped. Therefore, the tray position’s influence on the load sensor’s measured value was very much related to the air velocity.

### 3.2 Results and analyses of CNN training

#### 3.2.1 Selection of activation function and number of convolution kernels

The activation function and the number of convolutional kernels in the CNN needed to be determined by trial-and-error method based on the model performance index. The model performance test with different activation functions and number of convolution kernels was performed with the same training, validation, and test sets. The test results are shown in **Table 2**. **Table 2** shows that the model performance of the ReLU activation function was significantly better than that of the tanh and sigmoid functions. Li et al. (2022c) had similar findings when applying CNNs to predictive modeling. At the same activation function number (ReLU), a slight difference was found in the model performance for different numbers of convolutional kernels. The best model performance (*R^2^* closest to 1 and minimum RMSE) for the training, validation, and test sets occurred in the fourth, fifth, and fourth groups, respectively. The test set did not participate in the training process of the CNN, and its model performance was more reliable. The results of the test set of the fifth group were significantly worse than those of the fourth group. Taken together, the best structure of the CNN was the fourth group, the best activation function was ReLU, and the ideal number of convolutional kernels was (16, 32).

**Table 2:** Test results of different activation function and number of convolution kernels (shaded group is the optimal structure; bold font indicates the optimal solution).

Group	Activation function	Number of convolution kernels	Training set		Validation set		Test set	
			*R^2^*	*RMSE*	*R^2^*	*RMSE*	*R^2^*	*RMSE*
1	Relu	8, 16	0.9931	17.3	0.9860	31.4	0.9734	24.7
2		8, 32	0.9910	19.8	0.9888	22.1	0.9823	25.6
3		8, 64	0.9911	19.7	0.9892	21.7	0.9829	25.1
4		16, 32	**0.9957**	**13.8**	0.9876	23.2	**0.9913**	**17.9**
5		16, 64	0.9948	15.1	**0.9908**	**20.0**	0.9800	27.2
6		32, 64	0.9926	18.0	0.9867	24.1	0.9769	29.2
7	Tanh	8, 16	0.9314	54.7	0.9096	57.8	0.8710	75.0
8		8, 32	0.8904	69.2	0.8770	73.3	0.8795	66.8
9		8, 64	0.8892	53.4	0.8836	58.2	0.8854	58.0
10		16, 32	0.9439	49.5	0.8566	79.1	0.8625	71.3
11		16, 64	0.9377	40.9	0.9299	41.5	0.8947	56.8
12		32, 64	0.9248	57.3	0.8945	62.5	0.8891	69.5
13	Sigmoid	8, 16	0.9391	51.5	0.9229	53.4	0.9014	65.6
14		8, 32	0.9806	29.1	0.9749	33.1	0.9718	32.3
15		8, 64	0.9791	30.2	0.9644	39.4	0.9512	42.5
16		16, 32	0.9388	51.7	0.9214	53.9	0.8984	66.6
17		16, 64	0.9676	37.6	0.9630	40.2	0.9575	39.6
18		32, 64	0.9858	24.9	0.9714	35.3	0.9688	34.0

#### 3.2.2 Variable learning rate optimization

**Figure 6:**
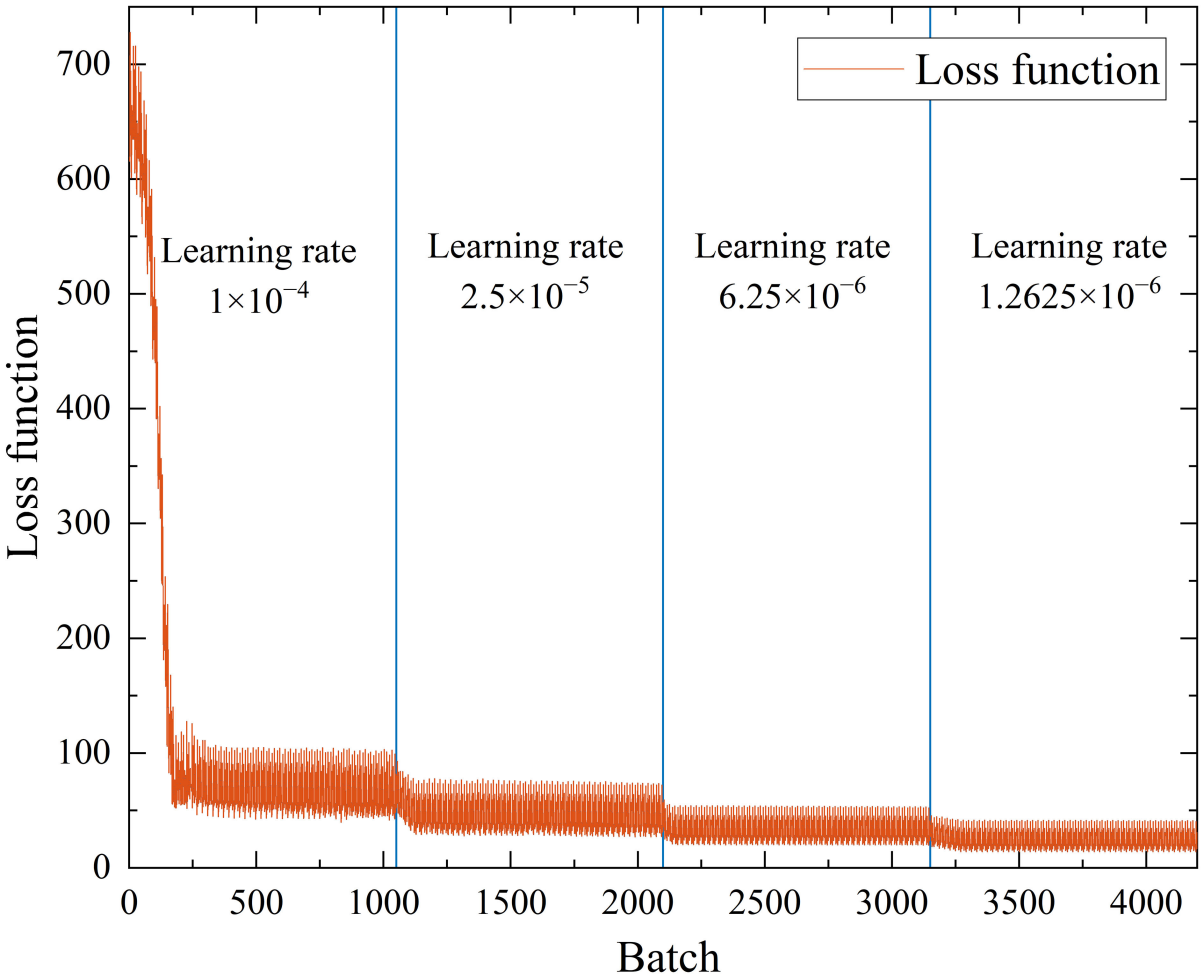
Loss function curve with variable learning rate optimization.

**Figure 7:**
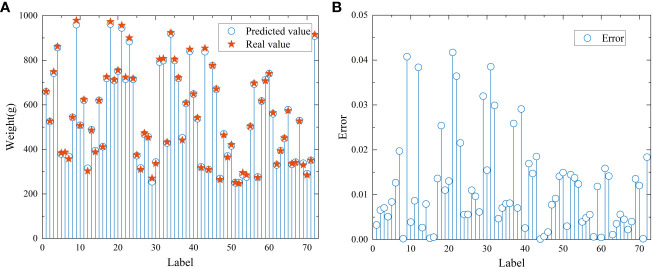
The prediction results of the CNN model test set. **(A)** and **(B)** are plots of fitting effects and prediction errors, in that order.

After the optimal activation function and number of convolution kernels were determined, the RMSE of the model was still high. An observation of the loss function curve during the training process of the CNN showed that the loss function showed regular oscillations at the late stage of training, but no decreasing trend occurred. This is the phenomenon of gradient disappearance caused by a too-large learning rate in the late training period (Noppitak and Surinta, 2022). However, if the learning rate was reduced, then the training time would be much longer, and obtaining the global optimum would be difficult. This study set the learning rate schedule to piecewise mode, which can adopt different learning rates in different training stages. The initial learning rate was set to 0.0001, the learning rate drop period was set to 50, the learning rate drop factor was set to 0.25, and the maximum number of iterations was set to 200. The loss function curve with variable learning rate optimization was shown in **Figure 6**. The maximum number of iterations was 200, the learning rate decreased every 50 iterations, and the loss function was updated 21 times per iteration (number of training set samples/batch size). The loss function was updated a total of 4200 times. In **Figure 6**, the loss function oscillation amplitude decreased with each decrease in the learning rate, which was due to gradient reduction caused by the decrease in the iteration step size. Every time the learning rate decreased, the loss function decreased significantly, and the whole training process showed a decreasing trend, which indicates that the iterative gradient was restored and the training results were constantly approaching the optimal value. The *R^2^* and RMSE of the model test set were 0.9989 and 6.9, respectively, and the prediction results of the model test set are shown in **Figure 7**. **Figures 7A, B** show the fitting degree and prediction error of the predicted value to the real value, respectively. The maximum error was 0.042, and 85% of the test data had a prediction error of less than 0.02. In conclusion, the prediction model with variable learning rate optimization can more accurately predict the material weight.

### 3.3 Model performance comparison

**Figure 8:**
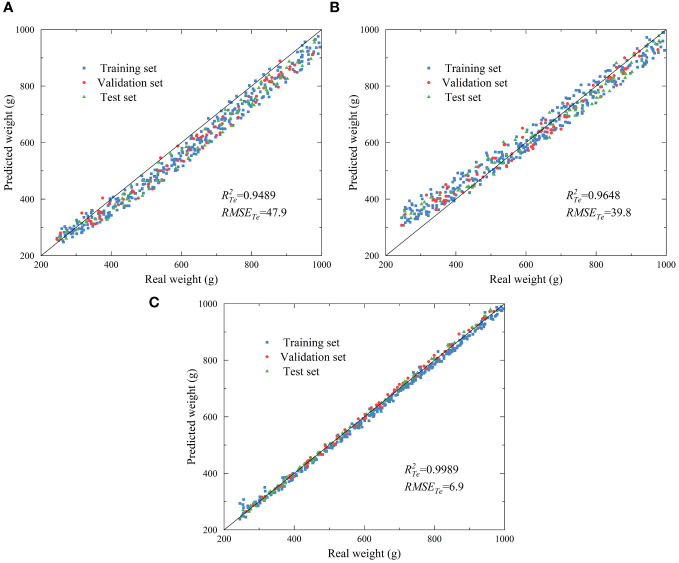
Scatterplot of PLSR **(A)**, SVM **(B)**, and CNN **(C)** models.

The CNN model is more complex than the other two prediction models, and the combination with hardware is much more difficult. Therefore, the performance of the classical linear and nonlinear regression models PLSR and SSVM was tested to prevent model performance excess. **Figures 8A–C** show scatterplots of real and predicted values for the PLSR, SVR, and CNN models. The solid line is a regression line that aids in analyzing the degree of deviation of the predicted values relative to the real values. The closer the scatter is to the regression line, the better the fit of the model. **Figure 8C** shows that the sample points of the CNN model were centrally distributed near the regression line, and the model had the best fit. In contrast, the PLSR and SVR model sample points were more dispersed, not exactly distributed near the regression line, and shifted relative to the regression line. The RMSE of the test set for the two models were 47.9 and 39.8, respectively, which cannot meet the accuracy requirements of moisture content online detection.

### 3.4 Validation test results

The moisture content detection based on the weighing method defaulted to the same initial moisture content of the same batch of material. A deviation occurred between the moisture content of the material used for the oven test and the drying experiment, which will inevitably affect the accuracy of moisture content detection. The validation test results are shown in **Table 3**. The RMSE of the five validation experiments was 1.47, which indicated that the error caused by defaulting to the same initial moisture content of the same batch of materials was within the acceptable range. The results of the five validation experiments showed that the fit of the moisture content detection model based on the CNN established in this study was acceptable, and the moisture content online detection system can accurately detect the moisture content of materials in the drying process.

**Table 3:** The validation test results.

	Group					*R^2^*	RMSE
	1	2	3	4	5		
Real moisture content	86.77%	81.38%	75.90%	64.46%	45.10%	0.9901	1.47
Predicted moisture content	86.58%	80.97%	74.99%	65.86%	47.90%		

## 4 Conclusion

In this study, a multi-sensor data acquisition platform was set up, and a CNN prediction model was established with raw monitoring data from the load sensor, air velocity sensor, temperature sensor and the tray position as inputs and the real weight of materials as outputs. The optimal activation function and number of convolutional kernels for the prediction model were selected. The optimal activation function was ReLU, and the optimal number of convolutional kernels was (16, 32). The training process of the CNN was optimized with a variable learning rate to optimize the model performance further. The final performance of the CNN prediction model was satisfactory (with *R^2^* and RMSE of 0.9989 and 6.9, respectively) and was significantly better than that of the traditional linear PLSA model (with *R^2^* and RMSE of 0.9489 and 47.9, respectively) and the nonlinear SVR model (with *R^2^* and RMSE of 0.9648 and 39.8, respectively). A moisture content online detection system was constructed based on the CNN prediction model. Validation experiments were carried out, and the *R^2^* and RMSE of the validation experiments were 0.9901 and 1.47, respectively. The validation experiments showed that the CNN prediction model was fully applicable to moisture content online detection, and the detection system based on this model fully met the accuracy requirements of moisture content online detection.

In the moisture content online detection system proposed in this study, the detection of initial moisture content still has errors. Future research can use more advanced and convenient technology to detect the initial moisture content of materials quickly and effectively. In addition, this system was built with computer as the host computer. Scholars can compile the detection model into the microcontroller in future research, which is conducive to the application and promotion of the detection system in actual production.

Overall, this study established a moisture content online detection system based on multi-sensor fusion and CNN prediction model, realizing real-time moisture content detection during agricultural products’ drying process. This study will provide technical support for further optimization of the drying process and will also promote the intelligent development of agricultural product drying equipment.

## Data availability statement

The raw data supporting the conclusions of this article will be made available by the authors, without undue reservation.

## Author contributions

TY: Conceptualization, Data curation, Formal analysis, Investigation, Methodology, Software, Validation, Visualization, Writing – original draft, Writing – review & editing. XZ: Conceptualization, Formal analysis, Investigation, Project administration, Supervision, Validation, Writing – review & editing. HX: Validation, Writing – review & editing. CS: Validation, Writing – review & editing. JZ: Validation, Writing – review & editing.

## References

[B1] Ari D. Alagoz B. B. (2022). An effective integrated genetic programming and neural network model for electronic nose calibration of air pollution monitoring application. Neural. Comput. Appl. 34, 12633–12652. doi: 10.1007/s00521-022-07129-0

[B2] Arvidsson S. Gullstrand M. Sirmacek B. Riveiro M. (2021). Sensor fusion and convolutional neural networks for indoor occupancy prediction using multiple low-cost low-resolution heat sensor data. Sensors 21, 1036. doi: 10.3390/s21041036 33546305 PMC7913583

[B3] Burnos P. Rys D. (2017). The effect of flexible pavement mechanics on the accuracy of axle load sensors in vehicle weigh-in-motion systems. Sensors 17, 2053. doi: 10.3390/s17092053 28880215 PMC5620507

[B4] Celik E. Parlak N. Cay Y. (2022). Development of an integrated corn dryer with an indirect moisture measuring system. Sadhana-Academy Proc. Eng. Sci. 47, 1. doi: 10.1007/s12046-021-01775-1

[B5] Chang A. Zheng X. Xiao H. Yao X. Liu D. Li X.. (2022). Short- and medium-wave infrared drying of cantaloupe (Cucumis melon L.) slices: drying kinetics and process parameter optimization. Processes 10, 114. doi: 10.3390/pr10010114

[B6] Cho J.-S. Choi J.-Y. Moon K.-D. (2020). Hyperspectral imaging technology for monitoring of moisture contents of dried persimmons during drying process. Food Sci. Biotechnol. 29, 1407–1412. doi: 10.1007/s10068-020-00791-x 32999748 PMC7492338

[B7] Dalvi-Isfahan M. (2020). A comparative study on the efficiency of two modeling approaches for predicting moisture content of apple slice during drying. J. Food Process Eng. 43, e13527. doi: 10.1111/jfpe.13527

[B8] Gao H. Li Y. Zhao Y. Song Y. (2023). Dual channel feature attention-based approach for RUL prediction considering the spatiotemporal difference of multisensor data. IEEE Sensors J. 23, 8514–8525. doi: 10.1109/jsen.2023.3246595

[B9] Guan Y. Meng Z. Sun D. Liu J. Fan F. (2022). Rolling bearing fault diagnosis based on information fusion and parallel lightweight convolutional network. J. Manufact. Syst. 65, 811–821. doi: 10.1016/j.jmsy.2022.11.012

[B10] Hu Y. Ma B. Wang H. Zhang Y. Li Y. Yu G. (2023). Detecting different pesticide residues on Hami melon surface using hyperspectral imaging combined with 1D-CNN and information fusion. Front. Plant Sci. 14, 1105601. doi: 10.3389/fpls.2023.1105601 37223822 PMC10200917

[B11] Ju H.-Y. Vidyarthi S. K. Karim M. A. Yu X.-L. Zhang W.-P. Xiao H.-W. (2023). Drying quality and energy consumption efficient improvements in hot air drying of papaya slices by step-down relative humidity based on heat and mass transfer characteristics and 3D simulation. Dry. Technol. 41, 460–476. doi: 10.1080/07373937.2022.2099416

[B12] Kirsanov D. Mukherjee S. Pal S. Ghosh K. Bhattacharyya N. Bandyopadhyay R.. (2021). A pencil-drawn electronic tongue for environmental applications. Sensors 21, 4471. doi: 10.3390/s21134471 34210087 PMC8272086

[B13] Li J. Zhou Q. Cao L. Wang Y. Hu J. (2022a). A convolutional neural network-based multi-sensor fusion approach for *in-situ* quality monitoring of selective laser melting. J. Manufact. Syst. 64, 429–442. doi: 10.1016/j.jmsy.2022.07.007

[B14] Li L. Xie S. Ning J. Chen Q. Zhang Z. (2019). Evaluating green tea quality based on multisensor data fusion combining hyperspectral imaging and olfactory visualization systems. J. Sci. Food Agric. 99, 1787–1794. doi: 10.1002/jsfa.9371 30226640

[B15] Li X. Jiang H. Liu Y. Wang T. Li Z. (2022b). An integrated deep multiscale feature fusion network for aeroengine remaining useful life prediction with multisensor data. Knowledge-Based Syst. 235, 107652. doi: 10.1016/j.knosys.2021.107652

[B16] Li Y. Ma B. Li C. Yu G. (2022c). Accurate prediction of soluble solid content in dried Hami jujube using SWIR hyperspectral imaging with comparative analysis of models. Comput. Electron. Agric. 193, 106655. doi: 10.1016/j.compag.2021.106655

[B17] Liu D. Zheng X. Xiao H. Yao X. Shan C. Chang A.. (2021). Optimization of sequential freeze-infrared drying process. Trans. Chin. Soc. Agric. Eng. 37, 293–302. doi: 10.11975/j.issn.1002-6819.2021.17.034

[B18] Ma T. Wang A. Dalca A. Sabuncu M. (2023). Hyper-convolutions *via* implicit kernels for medical image analysis. Med. Image Anal. 86, 102796. doi: 10.1016/j.media.2023.102796 36948069

[B19] Meng X. Zhang J. Xiao G. Chen Z. Yi M. Xu C. (2021). Tool wear prediction in milling based on a GSA-BP model with a multisensor fusion method. Int. J. Adv. Manufact. Technol. 114, 3793–3802. doi: 10.1007/s00170-021-07152-w

[B20] Noppitak S. Surinta O. (2022). dropCyclic: snapshot ensemble convolutional neural network based on a new learning rate schedule for land use classification. IEEE Access 10, 60725–60737. doi: 10.1109/access.2022.3180844

[B21] Pongsuttiyakorn T. Sooraksa P. Pornchalermpong P. (2019). Simple effective and robust weight sensor for measuring moisture content in food drying process. Sensors Mater. 31, 2393–2404. doi: 10.18494/sam.2019.2347

[B22] Reyer S. Awiszus S. Muller J. (2022). High-precision laboratory dryer for characterization of the drying behavior of agricultural and food products. Machines 10, 372. doi: 10.3390/machines10050372

[B23] Samaras S. Diamantidou E. Ataloglou D. Sakellariou N. Vafeiadis A. Magoulianitis V.. (2019). Deep learning on multi sensor data for counter UAV applications-A systematic review. Sensors 19, 4837. doi: 10.3390/s19224837 31698862 PMC6891421

[B24] Tan C. Li F. Lv S. Yang Y. Dong F. (2021). Gas-liquid two-phase stratified flow interface reconstruction with sparse batch normalization convolutional neural network. IEEE Sensors J. 21, 17076–17084. doi: 10.1109/jsen.2021.3081432

[B25] Tong J. Liu C. Zheng J. Pan H. (2023). Multi-sensor information fusion and coordinate attention-based fault diagnosis method and its interpretability research. Eng. Appl. Artif. Intell. 124, 106614. doi: 10.1016/j.engappai.2023.106614

[B26] Wan S. Li X. Zhang Y. Liu S. Hong J. Wang D. (2022). Bearing remaining useful life prediction with convolutional long short-term memory fusion networks. Reliabil. Eng. Sys. Saf. 224, 108528. doi: 10.1016/j.ress.2022.108528

[B27] Wang B. Lei Y. Li N. Wang W. (2021). Multiscale convolutional attention network for predicting remaining useful life of machinery. IEEE Trans. Ind. Electron. 68, 7496–7504. doi: 10.1109/tie.2020.3003649

[B28] Wang D. Li Y. Song Y. Jia L. Wen T. (2022). Bearing fault diagnosis method based on complementary feature extraction and fusion of multisensor data. IEEE Trans. Instrument. Measure. 71, 3527610. doi: 10.1109/tim.2022.3212542

[B29] Wang D. Lin H. Xiao H. Liu Y. Ju H. Dai J.. (2014). Design of online monitoring system for material moisture content in air-impingement drying process. Trans. Chin. Soc. Agric. Eng. 30, 316–324. doi: 10.3969/j.issn.1002-6819.2014.19.038

[B30] Wang D. Zhao F. Wang R. Guo J. Zhang C. Liu H.. (2023). A Lightweight convolutional neural network for nicotine prediction in tobacco by near-infrared spectroscopy. Front. Plant Sci. 14, 1138693. doi: 10.3389/fpls.2023.1138693 37251760 PMC10213436

[B31] Xie T. Huang X. Choi S.-K. (2022). Intelligent mechanical fault diagnosis using multisensor fusion and convolution neural network. IEEE Trans. Ind. Inf. 18, 3213–3223. doi: 10.1109/tii.2021.3102017

[B32] Xu X. Tao Z. Ming W. An Q. Chen M. (2020). Intelligent monitoring and diagnostics using a novel integrated model based on deep learning and multi-sensor feature fusion. Measurement 165, 108086. doi: 10.1016/j.measurement.2020.108086

[B33] Yang M. Liu N. Wu Y. Yang S. Yang L. Pu Y.. (2023a). Development and experiments of an online moisture content measuring device in thin layer hot-air drying process. Trans. Chin. Soc. Agric. Eng. 38, 47–56. doi: 10.11975/j.issn.1002-6819.202211176

[B34] Yang T. Zheng X. Vidyarthi S. K. K. Xiao H. Yao X. Li Y.. (2023b). Artificial neural network modeling and genetic algorithm multiobjective optimization of process of drying-Assisted walnut breaking. Foods 12, 1897. doi: 10.3390/foods12091897 37174434 PMC10178508

[B35] Yang T. Zheng X. Xiao H. Shan C. Ya1o X. Li Y.. (2023c). Drying temperature precision control system based on improved neural network PID controller and variable-temperature drying experiment of cantaloupe slices. Plants-Basel 12, 2257. doi: 10.3390/plants12122257 37375883 PMC10305149

[B36] Yu H. Hu Y. Qi L. Zhang K. Jiang J. Li H.. (2023). Hyperspectral detection of moisture content in rice straw nutrient bowl trays based on PSO-SVR. Sustainability 15, 8703. doi: 10.3390/su15118703

[B37] Zeng Y. Liu R. Liu X. (2021). A novel approach to tool condition monitoring based on multi-sensor data fusion imaging and an attention mechanism. Measure. Sci. Technol. 32, 055601. doi: 10.1088/1361-6501/abea3f

[B38] Zhao J. Ye X. Yue T. Li Y. (2023). CLDM: convolutional layer dropout module. Mach. Vision And Appl. 34, 63. doi: 10.1007/s00138-023-01411-4

[B39] Zheng Z. Wang S. Zhang C. Wu M. Cui D. Fu X.. (2023). Hot air impingement drying enhanced drying characteristics and quality attributes of ophiopogonis radix. Foods 12, 1441. doi: 10.3390/foods12071441 37048262 PMC10093796

[B40] Zhong Y. Teng Z. Tong M. (2023). LightMixer: A novel lightweight convolutional neural network for tomato disease detection. Front. Plant Sci. 14, 1166296. doi: 10.3389/fpls.2023.1166296 37229103 PMC10203629

[B41] Zhu J. Tang Y. Shao X. Xie Y. (2021). Multisensor fusion using fuzzy inference system for a visual-IMU-wheel odometry. IEEE Trans. Instrument. Measure. 70, 2505216. doi: 10.1109/tim.2021.3051999

